# Oral Administration of *Chaetoceros gracilis*—A Marine Microalga—Alleviates Hepatic Lipid Accumulation in Rats Fed a High-Sucrose and Cholesterol-Containing Diet

**DOI:** 10.3390/metabo13030436

**Published:** 2023-03-16

**Authors:** Bungo Shirouchi, Yuri Kawahara, Yuka Kutsuna, Mina Higuchi, Mai Okumura, Sarasa Mitsuta, Norio Nagao, Kazunari Tanaka

**Affiliations:** 1 Department of Nutrition Science, Faculty of Nursing and Nutrition, University of Nagasaki, Siebold, 1-1-1 Manabino, Nagayo-cho, Nishi-Sonogi-gun, Nagasaki 851-2195, Japan; 2 Blue Scientific Shinkamigoto Co., Ltd., 770 Kogushi, Shin-Kamigoto, Minami-Matsuura, Nagasaki 857-4601, Japan; 3 Regional Partnership Center, University of Nagasaki, Siebold, 1-1-1 Manabino, Nagayo-cho, Nishi-Sonogi-gun, Nagasaki 851-2195, Japan

**Keywords:** *Chaetoceros gracilis*, marine microalgae, fucoxanthin, protein source, hepatic lipid accumulation, lipogenesis, cholesterol absorption, metabolomics

## Abstract

Microalgae are attracting attention as a next-generation alternative source of protein and essential fatty acids that do not consume large amounts of water or land. *Chaetoceros gracilis* (*C*. *gracilis*)—a marine microalga—is rich in proteins, fucoxanthin, and eicosapentaenoic acid (EPA). Growing evidence indicates that dietary fucoxanthin and EPA have beneficial effects in humans. However, none of these studies have shown that dietary *C*. *gracilis* has beneficial effects in mammals. In this study, we investigated the effects of dietary *C*. *gracilis* on lipid abnormalities in Sprague-Dawley rats fed a high-sucrose cholesterol-containing diet. Dried *C. gracilis* was added to the control diet at a final dose of 2 or 5% (*w*/*w*). After four weeks, the soleus muscle weights were found to be dose-responsive to *C. gracilis* and showed a tendency to increase. The hepatic triglyceride and total cholesterol levels were significantly reduced by *C. gracilis* feeding compared to those in the control group. The activities of FAS and G6PDH, which are related to fatty acid de novo synthesis, were found to be dose-responsive to *C. gracilis* and tended to decrease. The hepatic glycerol content was also significantly decreased by *C. gracilis* feeding, and the serum HDL cholesterol levels were significantly increased, whereas the serum levels of cholesterol absorption markers (i.e., campesterol and β-sitosterol) and the hepatic mRNA levels of *Scarb1* were significantly decreased. Water-soluble metabolite analysis showed that the muscular contents of several amino acids, including leucine, were significantly increased by *C. gracilis* feeding. The tendency toward an increase in the weight of the soleus muscle as a result of *C. gracilis* feeding may be due to the enhancement of muscle protein synthesis centered on leucine. Collectively, these results show that the oral administration of *C. gracilis* alleviates hepatic lipid accumulation in rats fed a high-sucrose and cholesterol-containing diet, indicating the potential use of *C. gracilis* as a food resource.

## 1. Introduction

Numerous algae species are found worldwide and are classified into two groups—macroalgae (also known as seaweeds) and microalgae—depending on the complexity of their biological organization [1]. Microalgae are microscopic, unicellular, and photosynthetic organisms distributed in both freshwater and marine ecosystems [1,2]. They have attracted attention as promising candidates for the industrial exploitation of food and biofuels due to their high productivity per unit area compared to agricultural crops, their high adaptability to various cultivation conditions, and their capacity to grow rapidly [1,2]. From the perspective of sustainable food supply and security, microalgae are also attracting attention as next-generation alternative sources of protein and essential fatty acids that do not require the consumption of large amounts of water or land [2]. Currently, the majority of microalgae dominating the global market are freshwater species that are inexpensive and easy to grow, such as *Spirulina* and *Chlorella*, which can be produced in open ponds with little risk of contamination [3]. On the other hand, marine microalgae species are known to be efficient producers of bioactive compounds, such as carotenoids and n-3 polyunsaturated fatty acids [4]. Compared to fish oils, marine microalgae oils have several advantages, such as no unpleasant smell, less contamination with heavy metals, and no variations in fatty acid composition under controlled cultivation [5]. In Commission Implementing Regulation (EU) 2018/1023, the European Union’s list of novel foods was established to include several microalgae, including dried biomass and extracted oils [6].

Among the marine microalgae, *Chaetoceros gracilis* (*C. gracilis*) is classified as a diatom and is characterized by high protein, fucoxanthin, and eicosapentaenoic acid (EPA) contents, in addition to photosynthetic pigments such as chlorophyll a, *c*_1_, and *c*_2_ [4,7,8]. Fucoxanthin is a non-provitamin-A carotenoid that belongs to the xanthophyll family and has an allene structure. Fucoxanthin has been reported to have anti-proliferative and apoptosis-inducing effects on cancer cells [9,10], an anti-obesity effect [11], and an anti-diabetic effect [12,13], suggesting that the allene structure is involved in the expression of physiological functions. EPA is an essential fatty acid that humans are unable to produce and has both a lipid-lowering effect and an anti-inflammatory effect, leading to a lower risk of cardiovascular diseases [14,15]. Although *C. gracilis* is not currently included in the European Union’s list of novel foods, the intake of *C. gracilis*—which is rich in these bioactive compounds—is expected to have these beneficial effects.

To the best of our knowledge, no studies have reported the effects of *C. gracilis* as a dietary supplement against lipid abnormalities. Therefore, to explore the potential use of *C. gracilis* as a food resource, the present study examines the effects of dietary *C*. *gracilis* on lipid abnormalities in rats fed a high-sucrose, cholesterol-containing diet.

## 2. Experimental Design

### 2.1. Materials

*C. gracilis* was cultured, as described previously [7], at Blue Scientific Shinkamigoto Co., Ltd. (Nagasaki, Japan). Cultured *C. gracilis* was centrifuged at 7000 rpm at 25 °C at a flow rate of 0.8 L/min using a continuous high-speed centrifuge (H-660 type; KOKUSAN Co., Ltd., Saitama, Japan). Separated *C. gracilis* was freeze-dried for 3 days using a freeze dryer (EYELA FDU-1110; Tokyo Rikakikai Co., Ltd., Tokyo, Japan) and then stored at −20 °C until use. The nutritional composition of *C. gracilis* was analyzed by the Institute of Food Hygiene, the Nagasaki Food Hygiene Association, and the Food and Environment Research Center (Nagasaki, Japan) (Table 1). The compositions of amino acids [16] and fatty acids [17] in *C. gracilis* were also analyzed by the aforementioned institute. Their compositions are shown in Table S1 and Table S2, respectively. The pigments in *C. gracilis* were analyzed using a liquid chromatography mass spectrometer (LCMS), as previously described [7], and determined to be as follows (per g dry weight): 15.2 mg chlorophyll *a*, 5.73 mg chlorophyll *c*_1_, 0.868 mg chlorophyll *c*_2_, 11.8 mg fucoxanthin, 0.650 mg diadinoxanthin, and 0.455 mg diatoxanthin.

### 2.2. Animals and Diets

All animal experiments were conducted in accordance with the Guidelines for Animal Experiments of University of Nagasaki, Siebold, and Law no. 105 and Notification no. 6 of the government of Japan. The animal protocol used in this study was approved by the Institutional Review Board of University of Nagasaki, Siebold (authorization no. R02-12). Five-week-old male Sprague-Dawley rats (Jcl:SD) were purchased from CLEA Japan Inc. (Osaka, Japan). The rats were housed individually in metal cages in an air-conditioned room at 22 ± 1 °C and 55 ± 5% humidity under a 12 h light-dark cycle. After a one-week adaptation period on a powder chow diet (CE-2; CLEA Japan, Inc.), 19 rats were assigned to one of three groups (*n* = 6–7/group). The experimental diets were prepared according to the AIN-76 formula [18] with several modifications. Dried *C. gracilis* was added to the control diet at a final dose of 2 or 5% (*w*/*w*). The protein, fat, and other components (i.e., carbohydrate, ash, moisture, and sodium) in the diets containing *C. gracilis* were adjusted with casein, corn oil, and sucrose, respectively, to match the control diet. Diets containing high sucrose and cholesterol levels were used to induce lipid abnormalities. Further details regarding the composition of the experimental diets can be found in Table 2. The rats were provided free access to food and water for four weeks. Then, their feces were collected over a period of 48 h at the end of the experiment. At the end of the four-week feeding period and after a six-hour starvation period, the rats were euthanized by decapitation. To obtain serum, blood was incubated at room temperature for 30 min and then centrifuged at 1200× *g* for 15 min at 4 °C. The liver, soleus muscle, and abdominal (epididymal, perirenal, and mesenteric) white adipose tissue (WAT) and brown adipose tissue were excised immediately and weighed within 4 h. The collected samples were stored at −80 °C until further analysis.

### 2.3. Measurement of Serum Biochemical Parameters

The serum levels of triglycerides (TG), total cholesterol, phospholipids (PL), non-esterified fatty acids (NEFAs), glucose, and alanine aminotransferase (ALT) were measured using commercial enzyme assay kits (TG E-test Wako, Cholesterol E-test Wako, Phospholipid C-test Wako, NEFA C-test Wako, Glucose CII-test Wako, and Transaminase CII-test Wako; FUJIFILM Wako Pure Chemical Co., Osaka, Japan). Serum high-density lipoprotein (HDL) fractions were separated as described previously [19]. The serum cholesterol levels in the HDL fraction were measured using a commercial kit (Cholesterol E-test; Wako). The serum non-HDL cholesterol levels were calculated as the difference between the total cholesterol and HDL cholesterol levels. The serum levels of C-peptide, insulin, and adiponectin were measured using commercial rat ELISA kits (LBIS rat C-peptide ELISA kit and LBIS rat insulin ELISA kit; Shibayagi, Gunma, Japan; mouse/rat adiponectin ELISA kit; Otsuka Pharmaceutical, Tokyo, Japan).

### 2.4. Measurement of Triglycerides, Cholesterol, Phospholipids, and Glycogen Contents in the Liver

Total lipids from the liver were extracted using the Bligh and Dyer method, with some modifications, as described previously [20]. The extracted lipids were then dissolved in 2-propanol. The hepatic TG and total cholesterol contents were measured using commercial kits (TG E-test Wako and Cholesterol E-test Wako) [21]. The hepatic phospholipid content was measured according to the method described by Rouser et al. [22]. The hepatic glycogen content was measured according to the method described by Lo et al. [23].

### 2.5. Assays for Hepatic Enzyme Activities

The activities of fatty acid synthase (FAS), malic enzyme (ME), and glucose-6-phosphate dehydrogenase (G6PDH) in the cytosolic fraction, along with that of carnitine palmitoyltransferase (CPT) in the mitochondrial fraction, were determined as previously described [24]. The protein concentration of each fraction was determined according to the method described by Lowry et al. [25], using bovine serum albumin as the standard.

### 2.6. Measurement of Serum Steroid Levels

Biomarkers related to cholesterol metabolism—such as lathosterol, campesterol, and β-sitosterol [26,27]—in serum were measured by a gas chromatography-mass spectrometry (GC-MS) system using the Shimadzu GCMS-QP2010 Ultra (Shimadzu Corporation, Kyoto, Japan) equipped with an InertCap 5MS/NP capillary column (30 m × 0.25 mm i.d., 0.25 µm thickness, GL Sciences Inc., Tokyo, Japan) and using 5*α*-cholestane (Cayman Chemical Company, MI, USA) as an internal standard. Briefly, 250 µL of serum was added to 25 µg of 5*α*-cholestane. Then, the samples were saponified with ethanolic KOH and the steroids were extracted. The extracted steroids were converted to trimethylsilyl (TMS) ethers using the TMS derivatization reagent (*N*,*O*-Bis(trimethylsilyl)trifluoroacetamide (BSTFA)-trimethylchlorosilane (TMCS), (99:1); Tokyo Chemical Industry Co., Ltd., Tokyo, Japan) before injection into the GC-MS system. The following program was applied using helium as a carrier gas at a flow rate of 1.08 mL/min: 180 °C for 1 min, from 180 °C to 250 °C (20 °C/min), from 250 °C to 290 °C (5 °C/min), and 290 °C for 17.5 min. The total run time was 30 min. The injector was operated at a split ratio of 1:30 and was maintained at 300 °C. The interface temperature was 250 °C. The ion source temperature was 290 °C. The mass spectrometer was operated in the electron impact (EI) mode (70 eV). The mass scan range was 35–700 *m*/*z*.

### 2.7. Analysis of Hepatic mRNA Levels

Total RNA was extracted from frozen liver tissue using RNAzol^®^ RT reagent (Molecular Research Center, Inc., Cincinnati, OH, USA) with 4-bromoanisole (Molecular Research Center, Inc.) and converted to cDNA using PrimeScript^™^ RT Master Mix (Perfect Real Time) (Takara Bio Inc., Shiga, Japan), according to the manufacturer’s instructions. Polymerase chain reaction (PCR) amplification was performed in a final volume of 20 µL, which contained SYBR Green (THUNDERBIRD^®^ SYBR^®^ qPCR Mix; Toyobo Co., Ltd., Osaka, Japan), 0.3 µM of each primer (Fasmac Co., Ltd., Kanagawa, Japan), and 20 ng of cDNA, using a real-time PCR system (LightCycler^®^96; Nippon Genetics Co., Ltd., Tokyo, Japan). The reaction conditions were as follows: hot start at 95 °C for 60 s, followed by 45 cycles of denaturation at 95 °C for 15 s, and annealing/extension at 60 °C for 45 s. The relative mRNA levels were determined using the ΔΔCT method [28] using ribosomal protein L4 (*Rpl4*) as the housekeeping gene. The sequences of the primers used in this study are listed in Table S3.

### 2.8. Analysis of Water-Soluble Metabolites in the Liver and Soleus Muscle (Non-Target Metabolome Analysis)

Water-soluble metabolites in the liver and soleus muscle of rats in the two groups (control group and 5%*Chaeto* group) were analyzed according to the protocol described by Tsugawa et al. [29], with some modifications. Prior to metabolomic analysis, frozen liver (20 mg) or soleus muscle (50 mg) was homogenized using a ShakeMan 6 (Bio Medical Science, Tokyo, Japan) with zirconia beads. Homogenized samples were mixed with 250 µL of mixed solvent (methanol–H_2_O–chloroform = 5:2:2) and 10 µL of an internal standard solution (0.5 mg/mL) (2-isopropylmalic acid; Sigma-Aldrich Co., MO, USA). After vortexing, the samples were shaken using a shaking apparatus at 37 °C for 30 min. After centrifugation at 16,000× *g* and 4 °C for 3 min, the supernatants (180 µL) were collected, placed in new microtubes with 200 µL of H_2_O, and mixed using a vortex. The tubes were centrifuged at 16,000× *g* at 4 °C for 3 min. The resulting supernatants (250 µL) were collected into new microtubes for analysis.

To prepare quality control (QC) samples, 50 µL of each sample was mixed and dispensed into new tubes (200 µL/microtube). The analysis and QC samples were condensed using a centrifugal evaporator (EYELA CVE-2200; Tokyo Rikakikai Co., Ltd.) with a cold trap (EYELA UT-1010; Tokyo Rikakikai Co., Ltd.) at room temperature until the solvent evaporated. Methoxyamine hydrochloride (Sigma-Aldrich Co.) in anhydrous pyridine (20 mg/mL) was added to each tube, mixed using a vortex, and sonicated in an ice-cold water bath for 20 min with an ultrasonic cleaner. The mixture was shaken using a shaking apparatus for 90 min at 30 °C for oxidation. Next, 30 µL of *N*-methyl-*N*-trimethylsilyltrifluoroacetamine (MSTFA; GL Sciences Inc., Tokyo, Japan) was added to each tube and shaken with a shaking apparatus for 30 min at 37 °C to prepare the trimethylsilyl (TMS) derivatives. After centrifugation (16,000× *g*, room temperature, 5 min), the supernatant (80 µL) was collected into a 100 µL spitz attached to a 1.5 mL vial, which was sealed with a septum and a screw cap. The derivatized samples were analyzed with a GC-MS system (GCMS-QP2010 Ultra; Shimadzu) equipped with an InertCap 5MS/NP capillary column. The following program was applied using helium as a carrier gas at a flow rate of 1.12 mL/min: 80 °C for 2 min, from 80 °C to 330 °C (5 °C/min), and 330 °C for 6 min. The total run time was 58 min. The injector was operated at a split ratio of 1:20 and was maintained at 230 °C. The interface temperature was maintained at 250 °C. The ion-source temperature was maintained at 200 °C. The mass spectrometer was operated in EI mode (70 eV). The mass scan range was 85–500 *m*/*z*.

The obtained MS data (qgd-format) were converted to netCDF format. The data (netCDF format) were converted to the analysis base file (ABF) format by using an ABF converter (https://www.reifycs.com/AbfConverter/, accessed on 11 March 2023). Data processing was performed using MS-DIAL (version 4.92) [30,31]. Peak identification and prediction were also performed using MS-DIAL. The relative quantity of each metabolite was calculated using the peak area of each metabolite relative to that of an internal standard (2-isopropylmalic acid).

### 2.9. Statistical Analysis

All values are expressed as the mean ± standard error of the mean (SEM). All data were analyzed using Bartlett’s test (among three groups) or the *F*-test (between two groups) for equality of variance. When the data were recognized as unequal variances, they were normalized by logarithmic transformation and then re-analyzed using Bartlett’s test or the *F*-test. Parametric tests were used for further statistical analysis to confirm the homogeneity of all data. Comparisons among three groups (control, 2%*Chaeto*, and 5%*Chaeto* groups) were performed using one-way analysis of variance (ANOVA), followed by Dunnett’s multiple comparison post hoc test. The dose dependency of the *Chaeto* groups (2% and 5%) was examined using the Jonckheere–Terpstra trend test. Comparisons between two groups (control and 5%*Chaeto* groups) were performed using Student’s *t*-test for data with equal variances or Welch’s *t*-test for data with unequal variances. Results with *p* < 0.05 were considered statistically significant, and 0.05 ≤ *p* < 0.1 was considered a tendency.

Statistical analysis was performed using EZR, which is a graphical user interface for R (version 4.0.4) (The R Foundation for Statistical Computing, Vienna, Austria) [32], as well as MetaboAnalyst 5.0—a free web-based software platform [33]. Metabolites significantly affected by the 5%*Chaeto* diet compared to the control diet were subjected to pathway and enrichment analyses using MetaboAnalyst 5.0.

## 3. Results

### 3.1. Effects of C. gracilis Feeding on Morphometric Variables in SD Rats

**Figure 1:**
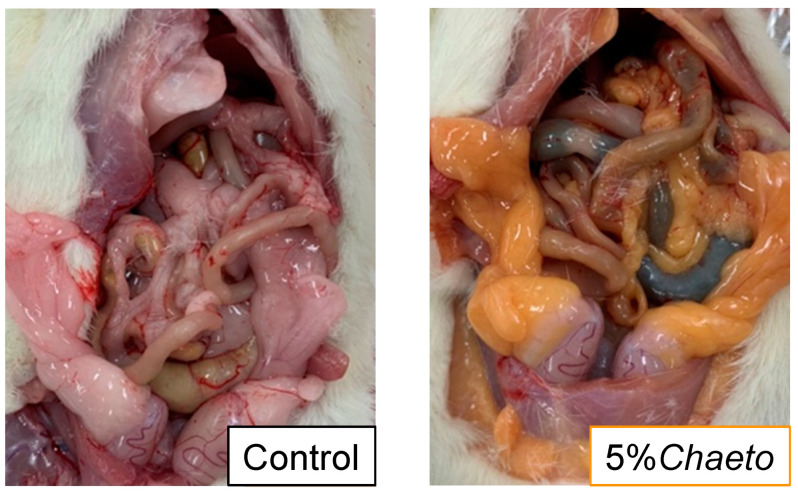
Macroscopic changes in the abdominal cavity of SD rats fed *C. gracilis*.

At the time of autopsy, the color of white adipose tissues of rats fed *C. gracilis* was found to turn “orange-like” (Figure 1). Table 3 summarizes the morphometric variables of SD rats after a four-week feeding period. Furthermore, no significant differences were observed in the final body weight, food intake, food efficiency, and organ or tissue weights—including the liver, several white adipose tissues, and brown adipose tissue—among the three groups. In contrast, dose dependency was observed in the weight of soleus muscle in the 2% and 5%*Chaeto* groups (*p* < 0.05, Jonckheere–Terpstra trend test). The soleus muscle weight tended to be higher in rats fed the 5%*Chaeto* diet than in those fed the control diet (*p* = 0.0916). Similarly, dose dependency was observed in the fecal weights in the 2% and 5%*Chaeto* groups (*p* < 0.05, Jonckheere–Terpstra trend test). Furthermore, a significant difference in fecal weight was observed between the control group and the 5%*Chaeto* group (*p* < 0.05, Dunnett’s multiple comparison test).

### 3.2. Effects of C. gracilis Feeding on Hepatic Lipid and Glycogen Contents and Serum Biochemical Parameters in SD Rats

Table 4 summarizes the hepatic lipid and glycogen contents and serum biochemical parameters in SD rats after a four-week feeding period. A dose dependency was observed in the hepatic TG contents in the 2% and 5%*Chaeto* groups (*p* < 0.05, Jonckheere–Terpstra trend test). The hepatic TG contents were significantly lower in the 2% and 5%*Chaeto* groups than in the control group. Dose dependency was also observed in the hepatic total cholesterol contents in the 2% and 5%*Chaeto* groups (*p* < 0.05, Jonckheere–Terpstra trend test). The hepatic total cholesterol content was significantly lower in the 5%*Chaeto* group than in the control group. However, no significant differences in hepatic PL and glycogen contents were observed among the three groups.

Dose dependencies were observed in the serum total cholesterol and HDL cholesterol levels in the 2% and 5%*Chaeto* groups (*p* < 0.05, Jonckheere–Terpstra trend test). The serum levels of total and HDL cholesterol were significantly higher in the 2% and 5%*Chaeto* groups than in the control group. In contrast, no significant differences were observed in the serum levels of TG, PL, NEFAs, glucose, C-peptide, insulin, adiponectin, or ALT among the three groups.

### 3.3. Effects of C. gracilis Feeding on Activities of Hepatic Enzymes and mRNA Levels Related to Fatty Acid Metabolism in SD Rats

Enzymatic activities related to hepatic fatty acid de novo synthesis and fatty acid β-oxidation in rats are summarized in Table 5. Dose dependencies were observed for the FAS and G6PDH activities in the 2% and 5%*Chaeto* groups (*p* < 0.05, Jonckheere–Terpstra trend test). FAS activity tended to be lower in rats fed the 5%*Chaeto* diet than in those fed the control diet (*p* = 0.0865). G6PDH activity was significantly lower in the 5%*Chaeto* group than in the control group. However, no significant differences were observed in the ME and CPT activities among the three groups. The hepatic mRNA levels of *Fasn* and *G6pd* did not differ significantly among the three groups (Table 5).

### 3.4. Effects of C. gracilis Feeding on Serum Levels of Steroids and Relative Levels of Hepatic mRNA Related to Cholesterol Metabolism in SD Rats

The serum steroid levels and relative levels of hepatic mRNA related to cholesterol metabolism in rats are summarized in Table 5. A dose dependency was observed in the serum levels of cholesterol absorption markers, such as campesterol and β-sitosterol in the 2% and 5%*Chaeto* groups (*p* < 0.05, Jonckheere–Terpstra trend test). The serum levels of campesterol and β-sitosterol were significantly lower in the 2% and 5%*Chaeto* groups than in the control group. In contrast, the serum levels of lathosterol—a cholesterol synthesis marker—did not significantly differ among the three groups. In addition, dose dependency was observed in the hepatic mRNA levels of *Scarb1*, known as the HDL receptor, in the 2% and 5%*Chaeto* groups (*p* < 0.05, Jonckheere–Terpstra trend test). The *Scarb1* mRNA levels were significantly lower in the 5%*Chaeto* group than in the control group. In contrast, the hepatic mRNA levels of *Hmgcr*, *Soat1*, and *Abca1* did not significantly differ among the three groups.

### 3.5. Effects of C. gracilis Feeding on Water-Soluble Metabolites in the Liver of SD Rats

Among the 66 metabolites identified and semi-quantified in the liver (Table S4), the levels of three metabolites (glycerol, hypotaurine, and inositol) were significantly reduced in the 5%*Chaeto* group compared to those in the control group (Table 6).

### 3.6. Effects of C. gracilis Feeding on Water-Soluble Metabolites in the Soleus Muscle of SD Rats

Among the 50 metabolites identified and semi-quantified in the soleus muscle (Table S5), the levels of 19 metabolites (2-aminoethanol, 3-hydroxypyruvate, β-alanine, cadaverine, creatine, glycerol, glycine, hypoxanthine, iminodiacetate, isoleucine, leucine, lysine, nicotinamide, *O*-phosphoethanolamine, phenylalanine, serine, threonine, uracil, and valine) were significantly increased, whereas the levels of one metabolite (oxalate) were significantly reduced in the 5%*Chaeto* group compared with those in the control group (Table 6).

**Figure 2:**
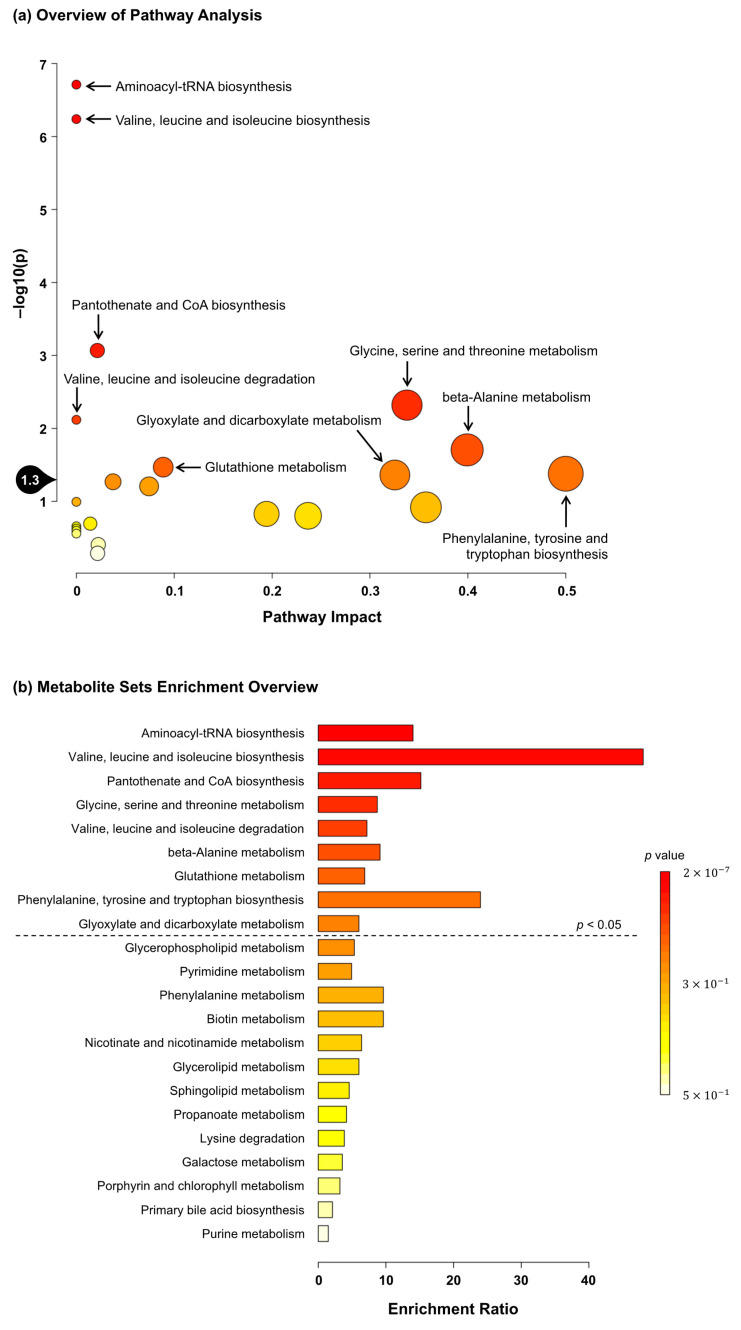
(**a**) Summary of MetaboAnalyst 5.0 pathway analysis for metabolites significantly different between the control and 5%*Chaeto* groups. The pathway impact on the *x* axis from 0 (low impact) to 1 (strong impact) represents the values from the pathway topology analysis. The circle diameter covaries with pathway impact, and the fill color intensity (white to red) reflects statistical significance (decreasing *p*-value). The black drop with 1.3 on the *y* axis indicates the threshold of significance at *p* < 0.05. (**b**) Summary of MetaboAnalyst 5.0 enrichment analysis for metabolites significantly different between the control and 5%*Chaeto* groups. The enrichment ratio is calculated as the number of hits within a particular metabolic pathway divided by the expected number of hits. Each bar shows a pathway, and the fill color intensity (white to red) reflects statistical significance (decreasing *p*-value). The pathways above the dotted line indicate significance at *p* < 0.05.

These 20 significant metabolites were subjected to pathway and enrichment analyses using MetaboAnalyst 5.0. The associated metabolic pathways are shown in Figure 2a, wherein nine pathways were found to be significantly related to *C. gracilis* feeding: “aminoacyl-tRNA biosynthesis,” “valine, leucine and isoleucine biosynthesis”, “pantothenate and CoA biosynthesis”, “glycine, serine and threonine metabolism,” “valine, leucine and isoleucine degradation”, “β-alanine metabolism”, “glutathione metabolism”, “phenylalanine, tyrosine and tryptophan biosynthesis,” and “glyoxylate and dicarboxylate metabolism”. Similarly, the enrichment ratio and *p*-values suggested that these nine pathways were significantly enriched (Figure 2b).

## 4. Discussion

To explore the potential use of *C. gracilis* as a food resource, the effects of dietary *C*. *gracilis* on lipid abnormalities were investigated in rats fed the high-sucrose and cholesterol-containing diet.

In a previous study that evaluated the safety of long-term administration of high-dose fucoxanthin (500 mg and 1000 mg/kg of body weight) in mice, increases in the levels of cholesterol and phospholipids in the blood, as well as in the weight of the liver were observed [34]. In the present study, the fucoxanthin intake calculated from its content in *C. gracilis* was approximately 40 mg/kg of body weight, and no findings other than increased serum total and HDL cholesterol levels were observed (Table 4). Although EPA has a TG-lowering effect [14], the intake of 2–5%*Chaeto* (including EPA) did not affect the serum TG levels (Table 4). In addition, the color of the white adipose tissues of rats fed *C. gracilis* was observed to turn “orange-like” (Figure 1). This was consistent with previous studies in which fucoxanthin was administered alone [34]. Hashimoto et al. showed that, in mice, dietary fucoxanthin undergoes metabolic conversion to amarouciaxanthin A in the liver via fucoxanthinol and preferentially accumulates as amarouciaxanthin A in the adipose tissue [35], suggesting that amarouciaxanthin A is involved in the orange coloration of the adipose tissue. Taken together, we believe that the fucoxanthin contained in *C. gracilis* contributed greatly to the outcomes of this study.

In the present study, the soleus muscle weights were found to be dose-responsive to *C. gracilis* and showed a tendency to increase (Table 3). Dietary intake of protein is known to be a prerequisite for the day-to-day maintenance of skeletal muscle mass, which stimulates an increase in muscle protein synthesis and attenuates muscle protein breakdown [36]. In a previous study, dietary fucoxanthin was found to protect against dexamethasone-induced muscle atrophy in mice [37]. Therefore, the intake of *C. gracilis*, which is rich in protein and fucoxanthin, may be effective in maintaining muscle mass.

As shown in Table 4, the hepatic TG content was significantly reduced in the 2% and 5%*Chaeto* groups compared with that in the control group. To understand the mechanisms underlying the hepatic TG-lowering action of *C. gracilis*, the activities of hepatic enzymes related to fatty acid metabolism were analyzed. Although the activities of CPT responsible for fatty acid β-oxidation in the mitochondria did not differ among the three groups, the activities of FAS and G6PDH—which are related to fatty acid de novo synthesis in the cytosol—were found to be dose-responsive to *C. gracilis* and showed a tendency to decrease (Table 5). The decreased activities of FAS and G6PDH were consistent with the results of a previous study in which mice were fed high-fat diets with fucoxanthin (0.05% and 0.2%) [38]. The hepatic mRNA levels of *Fasn* and *G6pd* did not differ among the three groups (Table 5), suggesting that the changes in FAS and G6PDH activities resulting from *C. gracilis* feeding represent post-translational regulation, but not transcriptional regulation. According to the results of metabolomic analysis in the liver, the glycerol content was significantly reduced in the 5%*Chaeto* group compared to that in the control group (Table 6). These results suggest that the reduction in hepatic TG content by *C. gracilis* feeding was attributable to the reduction in both fatty acids and glycerol, which are the substrates of TG.

As shown in Table 4, compared with the control group, the hepatic total cholesterol content was significantly reduced in the 5%*Chaeto* group. To gain insights into the effects of *C. gracilis* feeding on cholesterol metabolism, we analyzed the serum levels of cholesterol, biomarkers, and hepatic mRNA levels related to cholesterol metabolism. Lathosterol is a precursor of de novo cholesterol synthesis, and its serum levels can be used as an index of cholesterol synthesis in the body [26,27]. In addition, plant sterols—such as campesterol and β-sitosterol—are sterol isomers that cannot be synthesized in animal bodies, and their serum levels are positively correlated with cholesterol absorption rates [26,27].

Although the serum levels of lathosterol and the hepatic mRNA levels of *Hmgcr* did not differ among the three groups, the serum levels of campesterol and β-sitosterol were found to be dose-responsive to *C. gracilis* and showed a significant decrease (Table 4 and Table 5). Additionally, as shown in Table 4, the serum total cholesterol levels were significantly higher in the 2% and 5%*Chaeto* groups than in the control group. Higher total cholesterol levels were associated with significantly increased HDL cholesterol levels. A previous study showed that high-fat diets with fucoxanthin (0.05% and 0.2%) reduced the hepatic cholesterol content and HMG-CoA reductase activities and increased the plasma HDL cholesterol levels and fecal cholesterol contents in mice [38]. This behavior, except for cholesterol synthesis in the previous study, was consistent with the results of our study. This difference in cholesterol synthesis was thought to be due to the differences in animal species (rats in the present study versus mice in the previous study), dietary fat (10% fat based on corn oil in the present study versus 10% lard + 10% corn oil in the previous study), and cholesterol content. Unfortunately, the quality of the lard used in the diet of the previous study was unknown, and the cholesterol contents in the diets of the present and previous studies could not be accurately compared. In the present study, no significant difference in the serum non-HDL cholesterol levels was observed (Table 4), indicating that *C. gracilis* supplementation did not affect the secretion of cholesterol from the liver into the bloodstream. Beppu et al. reported that fucoxanthin intake increases the serum HDL cholesterol levels by decreasing the protein expression of SR-B1—a receptor of HDL—in the livers of mice [39]. As shown in Table 5, the hepatic *Scarb1* mRNA levels were significantly decreased in the 5%*Chaeto* group, supporting the results of a previous study. Taken together, these data suggest that the decreases in intestinal cholesterol absorption and HDL uptake from the bloodstream into the liver caused by C. *gracilis* feeding contribute to the reduction in hepatic total cholesterol content in rats. From the perspective of the utilization and safety of *C. gracilis* rich in fucoxanthin as a food resource, further studies using LDL animals—such as hamsters, which have a lipoprotein metabolism similar to that of humans—are needed to evaluate whether the increase in the total and HDL blood cholesterol levels is specific to HDL animals, such as rats and mice. The health benefits of n-3 PUFAs such as EPA and DHA on lipid metabolism are well known [14,15]. A systematic review of the differential effects of dietary EPA and DHA on cardiometabolic risk factors indicates that dietary DHA—but not EPA—increases blood HDL-cholesterol levels [40]. Thus, we consider that fucoxanthin (but not EPA) contained in *C. gracilis* contributes greatly to the changes in lipid metabolism observed in this study.

Since *C. gracilis* feeding tended to increase the soleus muscle weight of rats, water-soluble metabolite analysis in the soleus muscle was performed. As a result, compared to the water-soluble metabolite analysis in the liver, many metabolites in the muscle were significantly changed between the two groups (Table 6). The muscular contents of several amino acids, including branched-chain amino acids (BCAAs), were significantly increased in the 5%*Chaeto* group (Table 6). The magnitude of the muscle protein synthesis response to an ingested protein source is regulated at multiple levels, including dietary protein digestion and amino acid absorption, splanchnic amino acid retention, postprandial insulin release, transport, and uptake of amino acids into skeletal muscles [41]. The experimental diets used in this study were adjusted to ensure equal protein contents (Table 2). In terms of the amino acid contents of the 5%*Chaeto* diet, the contents of aspartic acid, glycine, alanine, arginine, and cystine were slightly higher and the contents of other amino acids were slightly lower compared to the control diet (Table S6). As the increase in the amino acid contents of muscles after *C. gracilis* feeding did not match the contents in the diet, this increase may be attributed to increases in protein digestion in *C. gracilis* and amino acid absorption, as well as amino acid uptake by the muscle. Leucine has been shown to upregulate the muscle protein synthesis machinery by activating the mechanistic target of the rapamycin complex 1 (mTORC1) signaling pathway [42]. Pathway and enrichment analyses associated with the significantly altered metabolites revealed that nine metabolic pathways were potentially affected by *C. gracilis* feeding. “Aminoacyl-tRNA biosynthesis” was one of the pathways found in the analysis (Figure 2a,b). Aminoacyl-tRNA synthetases are a family of essential enzymes used for protein synthesis that play pivotal roles in the ligation of tRNA with their cognate amino acids [43]. Therefore, these results suggest that the tendency toward an increase in the weight of the soleus muscle after *C. gracilis* feeding may be due to the enhancement of muscle protein synthesis centered on leucine. As several pathways that may be affected by *C. gracilis* feeding were identified in the present study, future studies should seek to focus on these pathways.

In conclusion, this study is the first to report that the oral administration of the marine microalga *C. gracilis* alleviates hepatic lipid accumulation in rats fed a high-sucrose and cholesterol-containing diet, indicating its potential use as a food resource. Through further comparative research with other marine microalgae containing bioactive compounds similar to those of *C. gracilis*, determining whether or not *C. gracilis* intake has a unique beneficial effect would be interesting. Although *C. gracilis* is rich in protein, its amino acid score is inferior to casein and egg white protein, which have scores of 100 [24], with tryptophan as the first limiting amino acid. Therefore, when considering *C. gracilis* as a dietary protein source, *C. gracilis* intake would ideally need to be combined with other protein sources.

## Figures and Tables

**Table 1:** Nutritional composition of *C. gracilis*.

Calories (kcal/100 g d.w.)	411
Moisture (g/100 g d.w.)	4.3
Protein (g/100 g d.w.)	48.4
Fat (g/100 g d.w.)	17.1
Carbohydrate * (g/100 g d.w.)	15.9
Ash (g/100 g d.w.)	14.3
Sodium (mg/100 g d.w.)	1199
Salt equivalent (g/100 g d.w.)	3.0

* Including dietary fiber; d.w., dry weight.

**Table 2:** Composition of experimental diets in the present study.

	Control	2%*Chaeto*	5%*Chaeto*
Ingredients		(g/kg diet)	
Sucrose	449	442.10	431.75
Casein	200	190.32	175.80
β-Cornstarch	150	150	150
Cellulose	50	50	50
Corn oil	100	96.58	91.45
*C*. *gracilis*	---	20	50
Mineral mixture (AIN-76)	35	35	35
Vitamin mixture (AIN-76)	10	10	10
DL-Methionine	3	3	3
Choline bitartrate	2	2	2
Cholesterol	1	1	1
Energy (kcal/kg diet)	3977	3967	3952

**Table 3:** Effects of *C. gracilis* feeding on morphometric variables of SD rats.

	Control	2%*Chaeto*	5%*Chaeto*	*p* for Trends ^#^
Initial body weight (g)	185 ± 3	186 ± 3	186 ± 3	N.S.
Final body weight (g)	428 ± 6	424 ± 12	419 ± 4	N.S.
Food intake (g/day)	24.8 ± 0.4	24.5 ± 0.8	24.1 ± 0.6	N.S.
Food efficiency (%) ^‡^	35.0 ± 0.6	34.5 ± 0.7	34.5 ± 0.3	N.S.
Organ weights (g/100 g of body weight)				
Liver	4.43 ± 0.13	4.44 ± 0.17	4.25 ± 0.11	N.S.
Soleus muscle	1.02 ± 0.03	1.09 ± 0.02	1.09 ± 0.03	*p* < 0.05
White adipose tissue (WAT) weights (g/100 g of body weight)				
Epididymal	1.33 ± 0.08	1.22 ± 0.03	1.30 ± 0.16	N.S.
Perirenal	1.59 ± 0.08	1.56 ± 0.14	1.54 ± 0.30	N.S.
Mesenteric	1.57 ± 0.09	1.39 ± 0.16	1.19 ± 0.18	N.S.
Abdominal ^†^	4.48 ± 0.18	4.17 ± 0.30	4.03 ± 0.63	N.S.
Brown adipose tissue (BAT) weight (g/100 g of body weight)				
	0.147 ± 0.007	0.182 ± 0.017	0.163 ± 0.014	N.S.
Feces weights (mg feces/g food intake)				
	81.1 ± 3.1	91.0 ± 4.9	104 ± 5 *	*p* < 0.05

Values represent the mean ± SEM (*n* = 6–7 per group). ^‡^ Food efficiency was calculated by applying the following formula: Food efficiency (%) = body weight gain (g)/food intake (g) × 100. ^†^ Abdominal WAT weights were calculated by summing the weights of the epididymal, perirenal, and mesenteric WAT. ^#^ Jonckheere–Terpstra trend test. * *p* < 0.05 (vs. control group) according to Dunnett’s multiple comparison test. N.S., not significant.

**Table 4:** Effects of *C. gracilis* feeding on hepatic lipid and glycogen contents and serum biochemical parameters in SD rats.

	Control	2%*Chaeto*	5%*Chaeto*	*p* for Trends ^#^
Hepatic contents of lipids and glycogen (mg/g liver)				
TG	49.8 ± 5.2	33.4 ± 5.6 *	18.8 ± 2.4 *	*p* < 0.05
T-Chol	8.29 ± 0.61	6.84 ± 0.72	4.26 ± 0.27 *	*p* < 0.05
PL	27.1 ± 0.7	27.2 ± 2.0	29.3 ± 1.3	N.S.
Glycogen	33.2 ± 6.1	27.3 ± 4.2	25.6 ± 4.4	N.S.
Serum biochemical parameters				
TG (mg/dL)	181 ± 21	156 ± 33	134 ± 24	N.S.
T-Chol (mg/dL)	95.0 ± 2.6	124 ± 4 *	138 ± 4 *	*p* < 0.05
HDL Chol (mg/dL)	43.9 ± 2.4	62.7 ± 5.0 *	80.8 ± 5.1 *	*p* < 0.05
Non-HDL Chol (mg/dL)	51.1 ± 2.6	61.5 ± 5.4	57.7 ± 4.9	N.S.
PL (mg/dL)	193 ± 10	213 ± 16	217 ± 7	N.S.
NEFAs (mmol/L)	0.609 ± 0.055	0.630 ± 0.060	0.654 ± 0.054	N.S.
Glucose (mg/dL)	131 ± 6	128 ± 6	121 ± 4	N.S.
C-peptide (pg/mL)	229 ± 68	222 ± 22	164 ± 23	N.S.
Insulin (pg/mL)	273 ± 134	141 ± 29	148 ± 26	N.S.
Adiponectin (µg/mL)	1.69 ± 0.15	1.54 ± 0.16	1.55 ± 0.15	N.S.
ALT (IU/L)	8.42 ± 0.69	7.64 ± 0.68	7.24 ± 0.45	N.S.

Values represent the mean ± SEM (*n* = 6–7 per group). ^#^ Jonckheere–Terpstra trend test. * *p* < 0.05 (vs. control group) according to Dunnett’s multiple comparison test. ALT, alanine aminotransferase; Chol, cholesterol; HDL, high-density lipoprotein; NEFAs, non-esterified fatty acids; N.S., not significant; PL, phospholipids; TG, triglycerides.

**Table 5:** Effects of *C. gracilis* feeding on activities of hepatic enzymes related to fatty acid metabolism, serum levels of steroids, and relative levels of hepatic mRNA related to cholesterol metabolism in SD rats.

	Control	2%*Chaeto*	5%*Chaeto*	*p* for Trends ^#^
Hepatic activities of enzymes related to fatty acid synthesis (mmol/min/mg protein)				
FAS	10.9 ± 0.6	9.22 ± 0.90	8.49 ± 0.96 ^(*p* = 0.0865)^	*p* < 0.05
G6PDH	35.0 ± 3.9	27.7 ± 8.2	18.9 ± 2.8 *	*p* < 0.05
ME	41.3 ± 4.8	38.3 ± 7.8	30.5 ± 4.3	N.S.
Hepatic activities of enzyme related to fatty acid β-oxidation (mmol/min/mg protein)				
CPT	5.34 ± 0.13	5.32 ± 0.23	5.05 ± 0.27	N.S.
Cholesterol synthesis marker in serum (mg/dL)				
Lathosterol	0.136 ± 0.028	0.163 ± 0.023	0.170 ± 0.024	N.S.
Cholesterol absorption markers in serum (mg/dL)				
Campesterol	5.41 ± 0.21	4.82 ± 0.14 *	4.34 ± 0.07 *	*p* < 0.05
β-Sitosterol	9.83 ± 0.45	7.04 ± 0.33 *	6.86 ± 0.15 *	*p* < 0.05
Relative levels of hepatic mRNA related to fatty acid synthesis and cholesterol metabolism (arbitrary unit)				
*Fasn*	1.00 ± 0.40	0.792 ± 0.350	0.452 ± 0.128	N.S.
*G6pd*	1.00 ± 0.08	1.21 ± 0.24	1.07 ± 0.21	N.S.
*Hmgcr*	1.00 ± 0.13	1.07 ± 0.15	0.971 ± 0.177	N.S.
*Soat1*	1.00 ± 0.07	1.14 ± 0.21	0.786 ± 0.199	N.S.
*Scarb1*	1.00 ± 0.06	0.886 ± 0.049	0.683 ± 0.105 *	*p* < 0.05
*Abca1*	1.00 ± 0.15	1.20 ± 0.13	1.06 ± 0.20	N.S.

Values represent the mean ± SEM (*n* = 6–7 per group). ^#^ Jonckheere–Terpstra trend test. * *p* < 0.05 (vs. control group) according to Dunnett’s multiple comparison test. *Abca1*, ATP-binding cassette protein A1; CPT, carnitine palmitoyltransferase; FAS, fatty acid synthase; G6PDH, glucose-6-phosphate dehydrogenase; *Hmgcr*, 3-hydroxy-3-methylglurary-CoA reductase; ME, malic enzyme; N.S., not significant; *Scarb1*, scavenger receptor class B member 1; *Soat1*, sterol *O*-acyltransferase 1.

**Table 6:** Effects of *C. gracilis* feeding on levels of identified water-soluble metabolites in the liver and soleus muscle of SD rats.

	Control	5%*Chaeto*
Levels of water-soluble metabolites in the liver (arbitrary unit ^#^)		
Glycerol	402 ± 22	239 ± 40 *
Hypotaurine	32.5 ± 5.3	10.6 ± 1.6 ^†^
Inositol	24.7 ± 1.3	17.6 ± 1.3 *
Levels of water-soluble metabolites in the soleus muscle (arbitrary unit ^#^)		
2-Aminoethnol	10.3 ± 0.7	17.8 ± 0.8 *
3-Hydroxypyruvate	194 ± 17	283 ± 18 *
β-Alanine	23.0 ± 3.0	54.8 ± 7.2 *
Cadaverine	17.6 ± 1.8	26.2 ± 3.5 *
Creatinine	52.7 ± 3.6	76.2 ± 3.7 *
Glycerol	189 ± 16	275 ± 14 *
Glycine	500 ± 69	727 ± 49 *
Hypoxanthine	39.5 ± 6.5	63.8 ± 8.6 *
Iminodiacetate	14.0 ± 2.2	28.5 ± 4.3 *
Isoleucine	72.6 ± 3.1	129 ± 14 ^†^
Leucine	142 ± 9	237 ± 27 ^†^
Lysine	154 ± 16	282 ± 45 *
Nicotinamide	31.0 ± 1.4	35.9 ± 1.6 *
*O*-Phosphoethanolamine	5.34 ± 0.41	7.59 ± 0.55 *
Oxalate	17.6 ± 1.4	13.1 ± 1.5 *
Phenylalanine	11.2 ± 1.2	23.4 ± 3.5 *
Serine	24.6 ± 2.5	51.0 ± 5.7 *
Threonine	23.4 ± 2.5	39.0 ± 3.7 *
Uracil	4.42 ± 0.61	7.11 ± 0.98 *
Valine	81.3 ± 3.3	142 ± 19 ^†^

Values represent the mean ± SEM (*n* = 6–7 per group). ^#^ The relative quantity of each metabolite was calculated using the peak area of each metabolite relative to an internal standard (2-isopropylmalic acid). * *p* < 0.05 (vs. control group) according to Student’s *t*-test. ^†^ *p* < 0.05 (vs. control group) according to Welch’s *t*-test.

## Data Availability

The data presented in this study are available upon request from the corresponding author. The data are not publicly available because a public archive platform has not yet been set up for data sharing.

## Supplementary Materials

The following supporting information can be downloaded at: https://www.mdpi.com/article/10.3390/metabo13030436/s1, Table S1: Amino acid contents of *C. gracilis*; Table S2: Fatty acid contents of *C. gracilis*; Table S3: Sequences of primers used for real-time PCR; Table S4: Levels of identified water-soluble metabolites in the liver of SD rats; Table S5: Levels of identified water-soluble metabolites in the soleus muscle of SD rats; Table S6: Contents of amino acids in experimental diets.
