# Supplementary material for: Oral Administration of Chaetoceros gracilis—A Marine Microalga—Alleviates Hepatic Lipid Accumulation in Rats Fed a High-Sucrose and Cholesterol-Containing Diet
Source: Metabolites. 2023 Mar 16;13(3):436. doi: 10.3390/metabo13030436 (PMC10051878; doi:10.3390/metabo13030436)
Supplement: Supplementary file 1 [file metabolites-13-00436-s001.zip › Shirouchi_TableS1_230312.pdf]

Table S1. Amino acid contents of *C. gracilis*.

Contents (mg/100 g d.w.)	
Aspartic acid	4140
Threonine	1830
Serine	2020
Glutamic acid	4200
Proline	1520
Glycine	1930
Alanine	2500
Valine	1790
Methionine	560
Isoleucine	1650
Leucine	2970
Tyrosine	1490
Phenylalanine	1860
Histidine	710
Lysine	2240
Arginine	2090
Cystine	790
Tryptophan	≤ 4

d.w., dry weight.
