# Supplementary material for: Oral Administration of Chaetoceros gracilis—A Marine Microalga—Alleviates Hepatic Lipid Accumulation in Rats Fed a High-Sucrose and Cholesterol-Containing Diet
Source: Metabolites. 2023 Mar 16;13(3):436. doi: 10.3390/metabo13030436 (PMC10051878; doi:10.3390/metabo13030436)
Supplement: Supplementary file 1 [file metabolites-13-00436-s001.zip › Shirouchi_TableS2_230312.pdf]

Table S2. Fatty acid contents of *C. gracilis*.

Contents (mg/100 g d.w.)	
Myristic acid (C14:0)	1352
Palmitic acid (C16:0)	2520
Heptadecanoic acid (C17:0)	425
Stearic acid (C18:0)	789
Palmitoleic acid (C16:1*n*-7)	3256
Oleic acid (C18:1*n*-9)	1157
Linolenic acid (C18:2*n*-6)	97
Arachidonic acid (C20:4*n*-6)	104
Eicosapentaenoic acid (C20:5*n*-3)	2535
Docosahexaenoic acid (C22:6*n*-3)	180

d.w., dry weight.
