# Supplementary material for: Oral Administration of Chaetoceros gracilis—A Marine Microalga—Alleviates Hepatic Lipid Accumulation in Rats Fed a High-Sucrose and Cholesterol-Containing Diet
Source: Metabolites. 2023 Mar 16;13(3):436. doi: 10.3390/metabo13030436 (PMC10051878; doi:10.3390/metabo13030436)
Supplement: Supplementary file 1 [file metabolites-13-00436-s001.zip › Shirouchi_TableS3_230312.pdf]

Table S3. Sequences of primers used for real-time PCR.

Gene name*	Accession no.	Direction	Primer sequence (5'→3')
Rpl4	NM_022510.1	Forward	CCGGAAGTTGGATGAGTTGT
		Reverse	TTTGCGTAAGGGTTCAGCTT
Fasn	NM_017332.2	Forward	TCAGCCCACAGGACAAGC
(synonym FAS)		Reverse	TCACCAAGCCTACCACACC
G6pd	NM_017006.2	Forward	GCAAACAGAGTGAGCCCTTC
(synonym G6PDH)		Reverse	TGGCTGTTGAGGTGCTTG
Hmgcr	NM_013134.2	Forward	TCTGCAGTACCTGCCTTACAGA
(synonym HMG-CoA reductase)		Reverse	ATCACGTTCTCACAGCAAGC
Soat1	NM_031118.1	Forward	GATGGGGTTATGTTGCTATGC
(synonym ACAT1)		Reverse	GGGCTCCTGTTTGATATTCCG
Scarb1	NM_031541.2	Forward	CCCTATGTCTACAGGGAGTTCA
(synonym SR-B1)		Reverse	CCTGTCTGGCTGGAAACGAA
Abca1	NM_178095.3	Forward	AGCAGTTTGTGGCCCTCTTG
(synonym ABCA1)		Reverse	AGTTCCAGGTTGGGGTACTT

*Each gene name is represented by the approved symbol.

Abca1, ATP-binding cassette protein A1; *Fasn*, fatty acid synthase; *G6ph*, glucose-6-phosphate dehydrogenase; *Hmgcr*, 3-hydroxy-3-methylglurary-CoA reductase; *Rpl4*, ribosomal protein L4; *Scarb1*, scavenger receptor class B member 1; *Soat1*, sterol *O*-acyltransferase 1.
