# Supplementary material for: Oral Administration of Chaetoceros gracilis—A Marine Microalga—Alleviates Hepatic Lipid Accumulation in Rats Fed a High-Sucrose and Cholesterol-Containing Diet
Source: Metabolites. 2023 Mar 16;13(3):436. doi: 10.3390/metabo13030436 (PMC10051878; doi:10.3390/metabo13030436)
Supplement: Supplementary file 1 [file metabolites-13-00436-s001.zip › Shirouchi_TableS4_230312.pdf]

Table S4. Levels of identified water-soluble metabolites* in the liver of SD rats.

	Control	5%*Chaeto*
Levels of water-soluble metabolites in the liver (arbitrary unit#)		
2-Aminobutyric acid	6.09 ± 0.80	5.47 ± 0.29
2-Deoxy-glucose	5.59 ± 2.25	7.11 ± 1.66
2-Hydroxybutyrate	3.65 ± 0.51	2.91 ± 0.52
4-Aminobutyric acid	22.7 ± 4.4	24.7 ± 2.8
4-Hydroxypyridine	0.258 ± 0.175	0.0125 ± 0.0072
5-Oxoproline	181 ± 14	165 ± 7
Alanine	367 ± 38	404 ± 51
Allose + Mannose	943 ± 44	925 ± 36
Arabitol	5.14 ± 0.54	4.17 ± 0.65
Aspartic acid	64.0 ± 12.4	72.8 ± 10.3
β-Alanine	6.34 ± 0.50	5.01 ± 0.86
β-Lactose	10.7 ± 1.0	11.1 ± 1.2
Fructose	11.7 ± 0.8	9.16 ± 1.94
Fumaric acid	4.90 ± 1.61	6.14 ± 1.75
Galactitol	6.28 ± 1.25	3.42 ± 0.71
Galactose	53.9 ± 4.1	46.6 ± 1.2
Galactose + Glucose	254 ± 8	251 ± 6
Galacturonic acid	5.24 ± 0.47	4.13 ± 0.58
Gentiobiose	2.13 ± 0.44	2.27 ± 0.44
Glucose	1063 ± 44	1039 ± 51
Glucuronate	2.51 ± 0.18	2.16 ± 0.12
Glutamic acid	105 ± 8	115 ± 18
Glutamine	5.45 ± 1.37	7.34 ± 3.04
Glyceric acid	2.48 ± 1.17	3.85 ± 2.02
Glycine	211 ± 9	187 ± 23
Hydroxyproline	11.1 ± 0.9	9.70 ± 0.75
Hypoxanthine	19.0 ± 1.3	17.6 ± 2.1
Iminodiacetate	67.5 ± 13.7	74.0 ± 10.1
Inosine	55.7 ± 7.2	49.5 ± 10.4
Isoleucine	45.8 ± 5.5	47.6 ± 6.3

Lactic acid	589 ± 34	609 ± 49
Lactitol	100 ± 12	114 ± 15
Leucine	118 ± 12	123 ± 16
Lysine	21.2 ± 2.5	23.4 ± 6.4
Malic acid	19.6 ± 5.7	24.4 ± 6.8
Maltose	262 ± 25	287 ± 36
Maltotriose	444 ± 35	450 ± 41
Melezitose	3.78 ± 0.27	3.69 ± 0.27
Methionine	12.8 ± 1.9	12.8 ± 1.9
O-Phosphoethanolamine	10.6 ± 1.0	10.3 ± 1.4
Ornithine	8.10 ± 0.40	6.21 ± 1.58
Oxalate	4.22 ± 1.04	6.88 ± 1.95
Phenylalanine	13.3 ± 1.6	14.1 ± 2.2
Phosphate	911 ± 20	939 ± 55
Phosphoenolpyruvic acid	7.13 ± 0.66	6.74 ± 1.29
Palmitic acid	13.8 ± 2.6	15.3 ± 1.1
Proline	19.8 ± 2.6	21.4 ± 5.8
Ribitol	7.13 ± 0.60	6.58 ± 0.34
Ribose	29.5 ± 5.7	30.0 ± 6.4
Serine	68.8 ± 6.8	72.6 ± 12.4
Sorbitol	6.13 ± 1.16	3.24 ± 0.75
Sorbose	10.4 ± 0.5	8.19 ± 1.70
Stearic acid	15.5 ± 2.9	17.5 ± 1.2
Taurine	20.6 ± 1.2	22.3 ± 1.6
Threonine	28.0 ± 2.1	27.4 ± 3.2
Trehalose	73.0 ± 6.9	81.3 ± 10.0
Tyrosine	42.7 ± 3.8	43.1 ± 5.5
Uracil	4.13 ± 0.89	6.00 ± 1.66
Uric acid	3.63 ± 2.45	4.17 ± 1.99
Uridine	17.1 ± 0.8	17.5 ± 0.8
Valine	61.8 ± 6.2	65.5 ± 9.7
Xanthine	29.2 ± 1.2	28.5 ± 2.9
Xylonic acid	3.19 ± 1.51	3.73 ± 0.89

Values are means ± SEM (n = 6–7 group).

[*]The 63 metabolites with CV% less than 10% are shown.

[#]Relative quantity of each metabolite was calculated using the peak area of each metabolite relative to an internal standard (2-isopropylmalic acid).
