# Supplementary material for: Oral Administration of Chaetoceros gracilis—A Marine Microalga—Alleviates Hepatic Lipid Accumulation in Rats Fed a High-Sucrose and Cholesterol-Containing Diet
Source: Metabolites. 2023 Mar 16;13(3):436. doi: 10.3390/metabo13030436 (PMC10051878; doi:10.3390/metabo13030436)
Supplement: Supplementary file 1 [file metabolites-13-00436-s001.zip › Shirouchi_TableS5_230312.pdf]

Table S5. Levels of identified water-soluble metabolites* in soleus muscle of SD rats.

	Control	5%*Chaeto*
Levels of water-soluble metabolites in soleus muscle (arbitrary unit#)		
2-Aminobutyric acid	11.6 ± 1.7	12.3 ± 3.0
2-Hydroxybutyrate	1.33 ± 0.49	1.30 ± 0.58
2-Hydroxypyridine	56.7 ± 3.8	56.2 ± 2.7
4-Methylbenzoic acid	1.38 ± 0.67	0.408 ± 0.045
5-Oxoproline	220 ± 18	265 ± 21
Alanine	759 ± 41	914 ± 195
Allose + Mannose	131 ± 9	127 ± 12
Fructose 6-phosphate	5.24 ± 2.89	1.54 ± 0.08
Fructose	6.23 ± 0.85	5.59 ± 1.68
Fumaric acid	17.2 ± 3.1	25.9 ± 2.5
Galactose + Glucose	32.3 ± 1.7	34.0 ± 1.5
Glucuronate	0.289 ± 0.232	0.441 ± 0.420
Glutamine	49.8 ± 5.6	73.6 ± 10.7
Hydroxyproline	5.07 ± 1.03	8.22 ± 1.18
Inosine 5'-phosphate	161 ± 9	187 ± 14
Inositol	18.9 ± 2.5	14.0 ± 1.5
Lactic acid	3656 ± 125	3981 ± 172
Malic acid	28.4 ± 5.1	39.2 ± 3.9
Oxamic acid	132 ± 22	149 ± 15
Phosphate	2348 ± 122	2612 ± 144
Palmitic acid	12.4 ± 2.6	9.07 ± 1.31
Proline	23.0 ± 3.7	36.2 ± 4.9
Stearic acid	17.2 ± 4.0	11.1 ± 1.8
Succinic acid	15.9 ± 3.6	14.0 ± 1.9
Taurine	455 ± 19	434 ± 33
Tryptophan	0.293 ± 0.042	2.71 ± 1.51
Tyrosine	90.5 ± 4.3	127 ± 19
Urea	158 ± 7	152 ± 18
Urocanate	29.8 ± 16.0	15.1 ± 9.2
Xanthine	10.6 ± 1.3	18.0 ± 3.8

Values are means ± SEM (n = 6–7 group).

[*]The 30 metabolites with CV% less than 10% are shown.

[#]Relative quantity of each metabolite was calculated using the peak area of each metabolite relative to an internal standard (2-isopropylmalic acid).
