# Supplementary material for: Oral Administration of Chaetoceros gracilis—A Marine Microalga—Alleviates Hepatic Lipid Accumulation in Rats Fed a High-Sucrose and Cholesterol-Containing Diet
Source: Metabolites. 2023 Mar 16;13(3):436. doi: 10.3390/metabo13030436 (PMC10051878; doi:10.3390/metabo13030436)
Supplement: Supplementary file 1 [file metabolites-13-00436-s001.zip › Shirouchi_TableS6_230312.pdf]

Table S6. Content of amino acids in experimental diets.

	Control	5%*Chaeto*
	(mg/kg diet)	
Aspartic acid	12600	13145
Threonine	8000	7947
Serine	10400	10152
Glutamic acid	38000	35502
Proline	20000	18340
Glycine	3400	3954
Alanine	5400	5997
Valine	12400	11795
Methionine	5400	5027
Isoleucine	10000	9615
Leucine	17000	16428
Tyrosine	10400	9887
Phenylalanine	9200	9017
Histidine	5400	5102
Lysine	14400	13778
Arginine	6800	7022
Cystine	880	1169
Tryptophan	2200	≦1936
